# Efficacy of as-needed intravitreal injection compared to 3-monthly loading of anti-vascular endothelial growth factor agents for branch retinal vein occlusion

**DOI:** 10.1038/s41598-023-39303-2

**Published:** 2023-07-26

**Authors:** Yoo-Ri Chung, Tae Kyoung Woo, Ha Ryung Park, Kihwang Lee

**Affiliations:** Department of Ophthalmology, Ajou University School of Medicine, 164 World Cup-Ro, Yeongtong-Gu, Suwon, 16499 Korea

**Keywords:** Diseases, Eye diseases

## Abstract

We investigated the efficacy of intravitreal injection of anti-vascular endothelial growth factor (VEGF) agents in branch retinal vein occlusion (BRVO). Databases, including PubMed, EMBASE, and the Cochrane Library, were searched on November 11, 2022. Studies comparing the pro-re-nata (PRN) regimen after the first treatment (PRN group) to three consecutive monthly injection regimens followed by the PRN regimen (3 + PRN group) were investigated. The primary outcomes were the change in best-corrected visual acuity (BCVA) and the change in central retinal thickness (CRT), with the secondary outcome being the injection frequency. Among 195 reports on anti-VEGF treatment, six comparative studies were included in this meta-analysis. The two groups had no statistically significant differences in terms of BCVA or CRT. However, the total number of injections during follow-up was significantly lower in the PRN group than in the 3 + PRN group (95% CI − 2.09 to − 0.83). The as-needed injection regimen is as effective as 3-monthly loading in terms of anatomical and functional improvement for BRVO, along with a lower treatment burden for patients and physicians.

## Introduction

Retinal vein occlusion (RVO) is a common retinal disorder that results in severe visual impairment^1^. Central retinal vein occlusion (CRVO) usually results in irreversible visual loss, especially in the ischemic type^1,2^, while the visual prognosis in branch retinal vein occlusion (BRVO) depends on various factors.

Macular edema (ME) is the main complication of RVO and requires treatment. Grid laser photocoagulation has been the treatment of choice for ME, while the introduction of various agents for intravitreal injections has changed the practice pattern^3^. Anti-vascular endothelial growth factor (VEGF) agents, including ranibizumab, bevacizumab, and aflibercept, were all effective in both functional and anatomical improvement in RVO^4^. Steroids such as dexamethasone implant or triamcinolone are also effective, while cataract progression and intraocular pressure elevation should be considered as possible adverse effects^4^. Consequently, intravitreal injections of anti-VEGF agents (IVT) are now the primary treatment modalities for ME due to RVO^4^.

Many studies have been performed to determine the optimal IVT frequency to preserve visual function in BRVO patients. Randomized controlled trials consisting of at least six monthly IVT of ranibizumab, such as the BRAVO and SHORE studies, have proven its efficacy^5,6^; however, these monthly injection protocols may lead to a significant burden for both patients and clinicians to be applied in clinical practice. The BRIGHTER study investigated the efficacy of 3-monthly IVT of ranibizumab, also showing significant visual improvement^7^. The MARVEL study showed that a protocol consisting of a single IVT followed by a pro-re-nata (PRN) strategy was also effective with either bevacizumab or ranibizumab^8^.

Although its efficacy has been reported in many studies, IVT always involves the potential risk of complications such as endophthalmitis or retinal detachment^9^. Moreover, injection frequency as a prognostic factor for vision remains controversial in BRVO^10^. Proactive loading of IVT ≥ 3 months may not be the most beneficial way to treat ME due to BRVO, considering that the as-needed protocol of IVT can be as effective in terms of anatomical and functional improvement with less frequent injection. Accordingly, we performed a meta-analysis of the efficacy of the PRN regimen, that is, as-needed regimen, after the first treatment compared to a 3-monthly loading injection followed by PRN for BRVO.

## Results

### Result of the literature search

**Figure 1:**
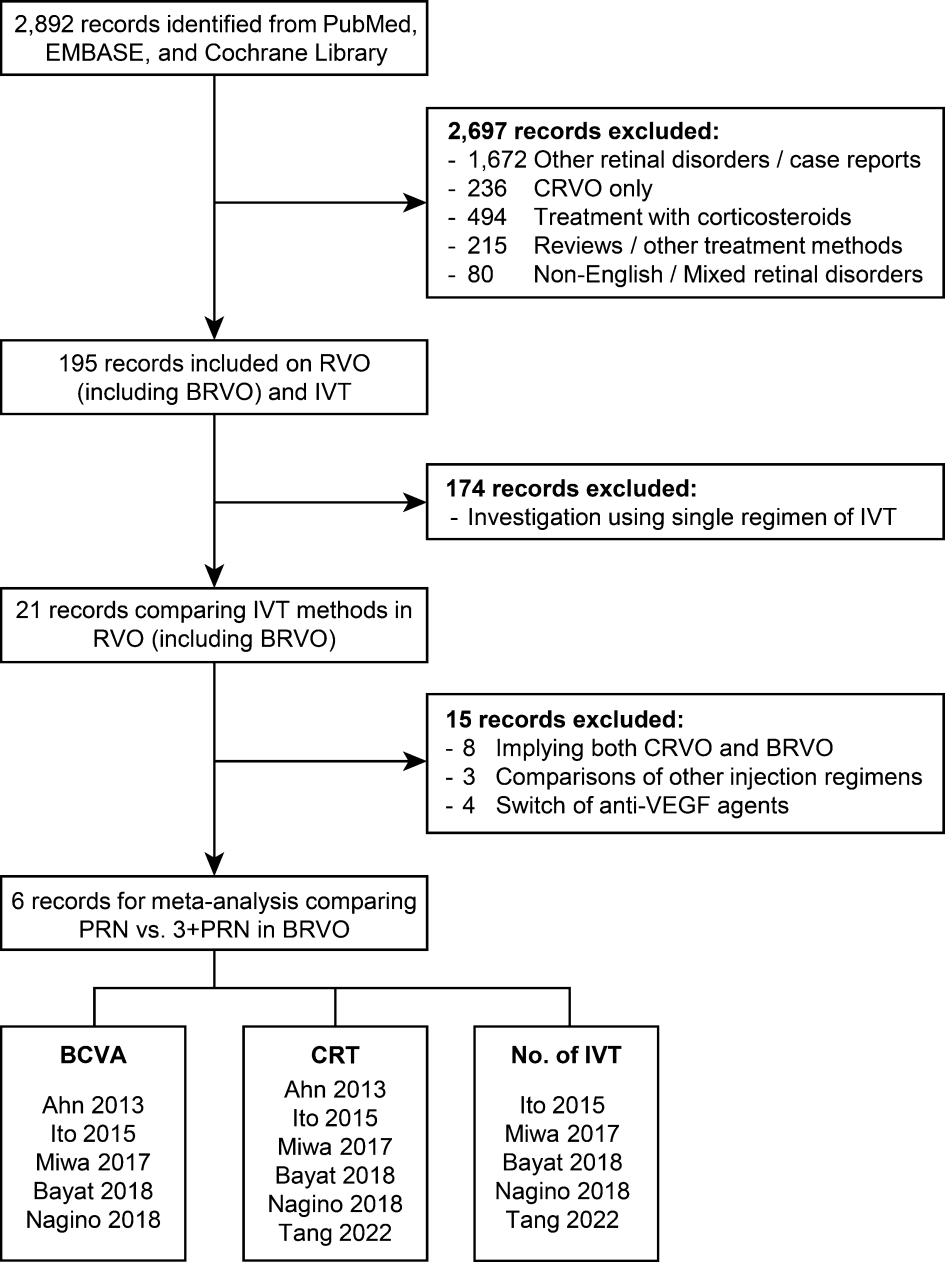
Flow diagram of the study selection process. *BCVA* best-corrected visual acuity, *BRVO* branch retinal vein occlusion, *CRT* central retinal thickness, *CRVO* central retinal vein occlusion, *IVT* intravitreal injection of anti-VEGF agents, *PRN* pro-re-nata, *RVO* retinal vein occlusion, *VEGF* vascular endothelial growth factor.

Among 195 reports regarding anti-VEGF treatment in patients with RVO, six comparative studies were finally included in this meta-analysis (Fig. 1). Among diverse regimens, we selected studies comparing the PRN regimen (as-needed injection) after the first treatment (“PRN” group) to 3- monthly loading injections followed by the PRN regimen (“3 + PRN” group).

There was one randomized controlled trial (RCT)^11^, two prospective interventional studies^12,13^, and three retrospective comparative case series^14–16^. Among anti-VEGF agents used in each study, three studies used ranibizumab^11,13,15^, two studies used bevacizumab^12,14^, and one study used either ranibizumab or aflibercept^16^. Details of the included studies are summarized in Table 1.

**Table 1:** Characteristics of included studies for meta-analysis.

Authors	Year, place	Study type (anti-VEGF)	Study groups (no. of eyes)	Study period	Change of BCVA (logMAR)	Change of CRT (μm)	No. of injections	Conclusion of the study
Ahn et al.^14^	2013, Korea	Retrospective, comparative case series (bevacizumab)	PRN (69) 3 + PRN (26)	6 months	PRN: − 0.26 ± 0.50* 3 + PRN: − 0.29 ± 0.26*	PRN: − 204 ± 356* 3 + PRN: − 161 ± 166*	PRN: 1.8 ± 0.8 3 + PRN: 3.4 ± 0.5	No significant differences in BCVA or CRT changes Fewer injections in PRN group
Ito et al.^12^	2015, Japan	Prospective, interventional study (bevacizumab)	PRN (25) 3 + PRN (27)	12 months	PRN: − 0.23 ± 0.23* 3 + PRN: − 0.29 ± 0.31*	PRN: − 250 ± 252* 3 + PRN: − 221 ± 233	PRN: 2.1 ± 0.8 3 + PRN: 4.3 ± 1.4	No significant differences in BCVA or CRT changes Fewer injections in PRN group
Miwa et al.^13^	2017, Japan	Prospective, interventional study (ranibizumab)	PRN (42) 3 + PRN (39)	12 months	PRN: − 0.25 ± 0.23 3 + PRN: − 0.29 ± 0.22	PRN: − 171 ± 156 3 + PRN: − 206 ± 155	PRN: 3.8 ± 1.8 3 + PRN: 4.6 ± 1.4	No significant differences in BCVA or CRT changes and number of IVT
Bayat et al.^15^	2018, Turkey	Retrospective, comparative case series (ranibizumab)	PRN (18) 3 + PRN (24)	12 months	PRN: − 0.33 ± 0.39 3 + PRN: − 0.50 ± 0.45	PRN: − 153 ± 175 3 + PRN: − 243 ± 160	PRN: 2.8 ± 1.6 3 + PRN: 4.2 ± 1.3	No significant differences in BCVA or CRT changes Fewer injections in PRN group
Nagino et al.^16^	2018, Japan	Retrospective, comparative case series (ranibizumab or aflibercept)	PRN (18) 3 + PRN (19)	12 months	PRN: − 0.24 ± 0.24 3 + PRN: − 0.29 ± 0.31	PRN: − 305 ± 193 3 + PRN: − 324 ± 168	PRN: 3.1 ± 1.6 3 + PRN: 5.1 ± 1.7	No significant differences in BCVA or CRT changes Fewer injections in PRN group
Tang et al.^11^	2022, China	Prospective, randomized controlled study (ranibizumab)	PRN (37) 3 + PRN (37)	12 months	PRN: 51.7 to 66.0^†^ 3 + PRN: 52.4 to 65.3^†^	PRN: − 300 ± 217 3 + PRN: − 298 ± 187	PRN: 4.2 ± 2.4 3 + PRN: 5.0 ± 2.2	No significant differences in BCVA or CRT changes and number of IVT

*BCVA* best-corrected visual acuity, *CRT* central retinal thickness, *IVT* intravitreal injection, *PRN* pro-re-nata, *VEGF* vascular endothelial growth factor.

*Calculated from the standard error and t value.

^†^ETDRS letters.

### Outcomes

**Figure 2:**
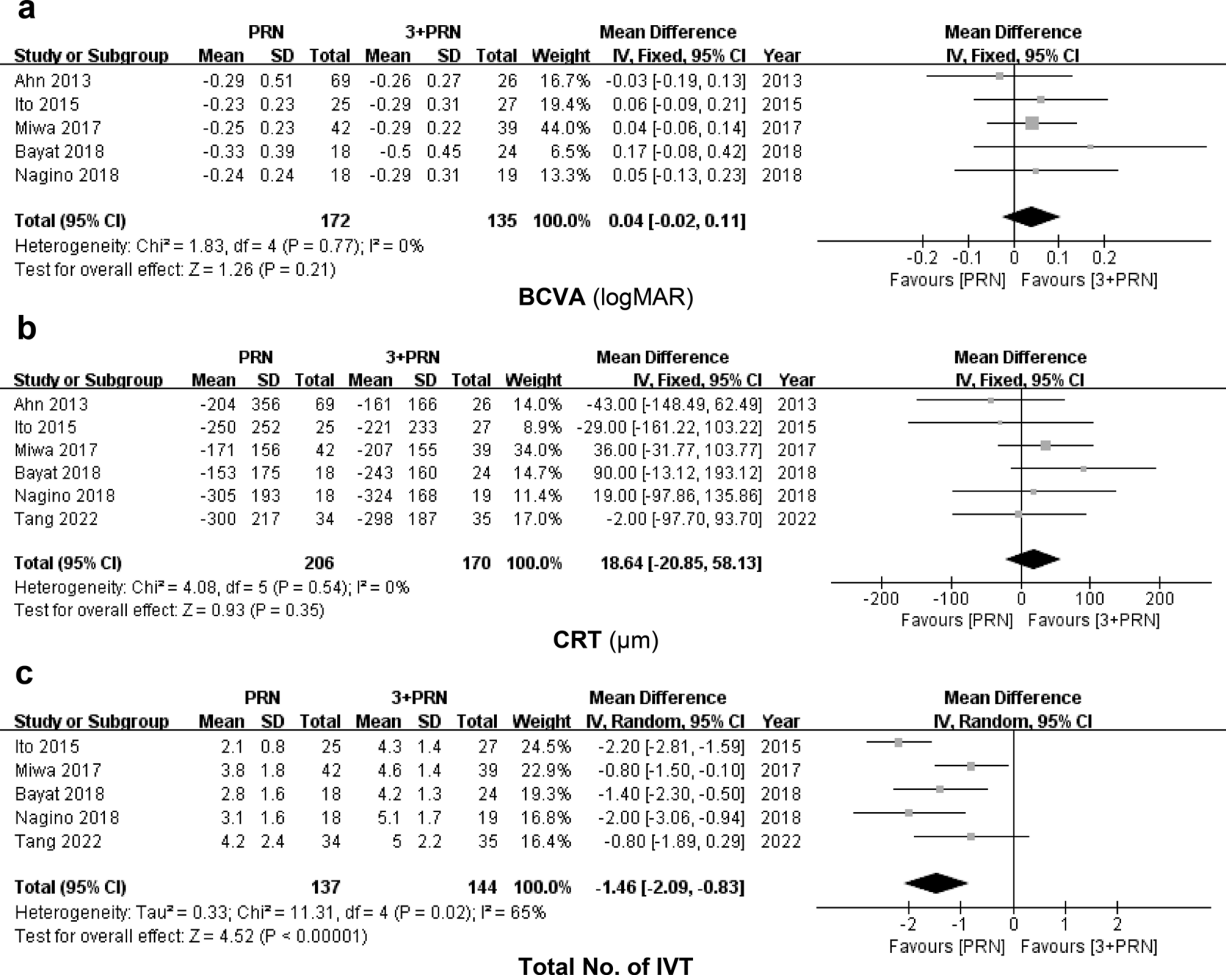
Forest plots of the mean differences in (**a**) best-corrected visual acuity by logMAR scale, (**b**) central retinal thickness, and (**c**) the total number of intravitreal injections with 95% confidence intervals comparing PRN group to 3 + PRN group.

For best-corrected visual acuity (BCVA), five studies with data presented by the logMAR scale were included in the meta-analysis, and all six studies were included for central retinal thickness (CRT). Each study presented no statistically significant preference in terms of BCVA between the two groups, resulting in a similar tendency in the meta-analysis (95% CI − 0.02 to 0.11, *P* = 0.21; Fig. 2a). This tendency was similar to that of CRT (95% CI, − 20.85 to 58.13; *P* = 0.35; Fig. 2b).

However, the total number of injections for 12 months of follow-up was significantly lower in the PRN group than in the 3 + PRN group (95% CI − 2.09 to − 0.83, *P* < 0.001, Fig. 2c).

### Sensitivity analysis

Sensitivity analysis was performed, excluding the results of Ahn et al.^14^, where the study duration was half that of other studies and that of Nagino et al.^16^, whose quality was assessed to be poor. The study by Tang et al.^11^ was additionally excluded from the sensitivity analysis of BCVA due to different units.

Sensitivity analyses of BCVA, CRT, and injection frequency showed similar results. There were no significant preferences for either treatment regimen in terms of BCVA (95% CI − 0.02 to 0.14) and CRT (95% CI − 15.56 to 75.90), while the total number of IVT was significantly lower in the PRN group (95% CI − 2.09 to − 0.60). Forest plots of these sensitivity analyses are presented in the Additional File 1.

### Quality assessment

The prospective RCT by Tang et al.^11^ assessed data using the Delphi list, scoring 6 points that met the following criteria: randomization, baseline group similarity, eligibility criteria specified, blinded outcome assessor, point estimates presented, and intention-to-treat analysis. The risk of bias for other studies using the NHLBI tool is presented in Table 2.

**Table 2:** Quality assessment of case–control studies using NHLBI tool.

Authors	Year, place	Q1	Q2	Q3	Q4	Q5	Q6	Q7	Q8	Q9	Q10	Q11	Q12	Quality
Ahn et al.^16^	2013, Korea	Yes	Yes	NR	Yes	Yes	Yes	NA	CD	Yes	Yes	NA	No	Fair
Ito et al.^14^	2015, Japan	Yes	Yes	NR	Yes	Yes	Yes	NA	CD	Yes	Yes	NR	No	Fair
Miwa et al.^15^	2017, Japan	Yes	Yes	NR	Yes	Yes	Yes	NA	No	Yes	Yes	NR	No	Fair
Bayat et al.^17^	2018, Turkey	Yes	Yes	NR	Yes	Yes	Yes	NA	CD	Yes	Yes	NR	Yes	Fair
Nagino et al.^18^	2018, Japan	Yes	Yes	NR	Yes	CD	CD	NA	CD	Yes	Yes	NR	No	Poor

For detailed criteria, please check the NHLBI site (https://www.nhlbi.gov/health-topics/study-quality-assessment-tools).

*CD* cannot determine, *NA* not applicable, *NR* not reported.

## Discussion

ME is the most common cause of vision loss in patients with BRVO. Intravitreal anti-VEGF has become the first-line therapy for ME due to BRVO based on the remarkable therapeutic effects shown in many prospective studies^4^. Regarding the dosing regimen, the SHORE study revealed that an as-needed regimen with monthly follow-up after seven monthly injections was as effective as a monthly fixed treatment regimen^6^. The BRIGHTER study confirmed the long-term efficacy of three loading injections followed by PRN dosing regardless of the degree of retinal ischemia and disease duration^17^. The MARVEL study that had an PRN regimen after the first injection showed comparable VA results to the BRAVO study at month 6 with a statistically significantly lower mean number of injections^8^. Their study may suggest reduced treatment burden but had limitations due to the small number of patients and short follow-up^8^. However, the result of the MARVEL study could be further supported based on our meta-analysis.

The recurrence of ME in BRVO, that is, the need for retreatment, relies on various factors. Many investigators have attempted to identify the prognostic factors associated with recurrence. Patients with BRVO with a higher risk of ME recurrence might need to be identified, as they require more intensive IVT treatment. Various risk factors exist for the recurrence of ME in BRVO, including baseline vision, area/size of the non-perfusion area, reperfusion state, and underlying medical factors^1^. Early treatment associated with shorter duration of symptom and better baseline visual acuity resulted in significantly less recurrence of ME^18,19^. Anatomical factors available on optical coherence tomography (OCT) were as follows: choroidal thickness, baseline CRT, presence of disorganized retinal inner layer or outer layer, and thickness of the parafoveal inner retina^18,20–22^. Non-perfusion of more than half of the 1 mm zone of the Early Treatment of Diabetic Retinopathy Study (ETDRS) circle was a factor predictive of fluorescein angiography finding for the recurrence of ME^22^. The introduction of OCT angiography also provided several potential prognostic factors, such as the destruction of the perifoveal capillary ring and central/paracentral non-perfusion area of the superficial capillary plexus^23,24^. Longer pre-treatment duration, short occlusion distance from the optic disc, thick CRT, and external limiting membrane disruption were associated with refractory ME in BRVO^25^. The lists of various risk factors for ME recurrence support the idea of an individualized approach to each patient based on numerous baseline features and treatment response, minimizing the overtreatment to lower the treatment burden for patients and physicians. Therefore, an as-needed IVT regimen after the first treatment is recommended, and our results also support this treatment regimen as effective as 3-monthly loading in terms of anatomical and functional improvement.

There were also studies investigating the treatment pattern of clinicians in the real world^26–28^. The survey on treatment patterns for BRVO from Japan revealed that an initial single injection followed by an as-needed IVT regimen was favored by more than 80% of clinicians, even in severe cases with BCVA < 0.1^27^. In contrast, 68.7% of the retina specialists from the American Society of Retina Specialists membership database out of 20% who participated in the survey chose a loading dose followed by a PRN injection without mentioning the specific number of loading^26^. The percentage of clinicians initiating IVT as single injection was similar to that of 3-monthly injections in one survey with RVO from Spain^28^. These findings suggest that the optimal IVT frequency remains controversial in clinical practice. This meta-analysis showed that the efficacy of as-needed IVT was not inferior to 3-monthly loading in terms of anatomical and functional outcomes, while the injection frequency was significantly reduced.

This study had several limitations. There was only one RCT among the included studies, and the other studies were not randomized. The number of patients in each study used for the meta-analysis was relatively small. The clinical heterogeneity among the studies was considerable in several aspects, including symptom duration before treatment, anti-VEGF agents used for IVT, inclusion criteria for eligibility, and retreatment criteria. This heterogeneity of anti-VEGF agents is especially crucial in the aspect of the injection frequency, as aflibercept and bevacizumab have longer intravitreal half-lives than ranibizumab^29,30^. However, real-world studies comparing between anti-VEGF agents in PRN method revealed no significant differences in the number of IVT^8,31,32^, suggesting that the injection frequency in clinical practice does not always correlate with pharmacokinetics of anti-VEGF agents. The different retreatment criteria should be also considered in interpreting the results of injection frequency in the meta-analysis. Some studies used OCT guided retreatment such as the increase in CRT or the presence of intraretinal/subretinal fluid, while there were studies that also considered BCVA as well. To note, each included study applied the same retreatment criteria for both PRN and 3 + PRN groups, mostly showing lower injection frequency in PRN group respectively. Although the details of retreatment criteria were not identical among studies, the meta-analysis can provide average outcomes representative for broad studies with improved accuracy. The lack of detailed features of BRVO such as the extent of ischemic area, which can affect the recurrence of ME, is another limitation of this study. Language bias also exists as the eligible studies were written in English.

In conclusion, the PRN regimen after the first treatment is as effective as a 3-monthly loading injection followed by PRN in terms of anatomical and functional improvement, along with a lower treatment burden for patients and physicians.

## Methods

### Search method

Databases including PubMed, EMBASE, and Cochrane library were last searched on November 11, 2022, implying the following terms for keywords: ‘retinal vein occlusion’, ‘macular edema’, ‘intravitreal injection’, ‘bevacizumab’, ‘ranibizumab’, ‘aflibercept’, ‘visual acuity’, ‘central foveal thickness’, ‘central retinal thickness’, and ‘central macular thickness’. A total of 2,892 studies were identified via a preliminary search. A further exclusion was performed based on the following criteria: (1) studies involving diabetic macular edema or age-related macular degeneration; (2) studies implying CRVO only; (3) using steroids (triamcinolone or dexamethasone) as intravitreal agents; (4) treatment other than intravitreal injection such as laser photocoagulation or oral medication; (5) case reports or review articles; and (6) duplicated articles and those written in non-English languages.

### Outcomes

Data on the mean values and standard deviations were obtained from the literature. The primary outcomes were changes in BCVA and CRT. Since there were studies with different follow-up periods, changes in BCVA or CRT from baseline to the last visit were included. The secondary outcome was injection frequency, which was presented as the total number of IVT during the study period.

### Quality assessment

The risk of bias among the included studies was assessed using the study quality assessment tool developed by the National Heart, Lung, and Blood Institute (NHLBI, https://www.nhlbi.gov/health-topics/study-quality-assessment-tools). Two reviewers (YRC and KL) assessed each individual criterion of the NHLBI tool for case–control studies and then discussed the overall judgment of quality as good, fair, or poor.

For the randomized study, the Delphi list was used for quality assessment^33^. Consisting of a total of nine criteria. One point was awarded for each criterion: randomization, treatment allocation concealment, baseline group similarity, eligibility criteria specified, blinded outcome assessor, care provider blinded, patient blinded, point estimates presented, and intention-to-treat analysis.

### Sensitivity analysis

Sensitivity analysis was performed, including studies with the same study periods and low-to-moderate risks of bias (i.e., fair quality). Accordingly, one study presented as a congress abstract and another with a shorter follow-up period were excluded from sensitivity analysis. Three studies were finally included for BCVA, and four studies for CRT and injection frequency.

### Statistical analysis

Data on the mean values and standard deviations were obtained from the literature. If the data for standard deviations were not presented in the literature, they were calculated from the standard error and t-value^34^. The meta-analysis was conducted using RevMan 5.4. Heterogeneity was examined using *I*^2^ statistics. The fixed effects model was applied when *I*^2^ was less than 50%, while the random effects model was applied when *I*^2^ was more than 50%. Squares indicate mean difference estimates, and lines extending from the squares represent the associated 95% confidence intervals in the forest plot. Confidence intervals that do not intersect the vertical line at 0 indicate statistical significance at the 0.05.

## Supplementary Information

Supplementary Information.

## Data Availability

The datasets supporting the conclusions of this article are included within the article and its additional file.
