# Supplementary material for: Efficacy of as-needed intravitreal injection compared to 3-monthly loading of anti-vascular endothelial growth factor agents for branch retinal vein occlusion
Source: Sci Rep. 2023 Jul 26;13:12068. doi: 10.1038/s41598-023-39303-2 (PMC10372086; doi:10.1038/s41598-023-39303-2)
Supplement: Supplementary file 1 — Supplementary Information. [file 41598_2023_39303_MOESM1_ESM.pdf]

a

Study or Subgroup	PRN Mean	PRN SD	PRN Total	3+PRN Mean	3+PRN SD	3+PRN Total	Weight	Mean Difference IV, Fixed, 95% CI	Year
Ito 2015	-0.23	0.23	25	-0.29	0.31	27	27.7%	0.06 [-0.09, 0.21]	2015
Miwa 2017	-0.25	0.23	42	-0.29	0.22	39	62.9%	0.04 [-0.06, 0.14]	2017
Bayat 2018	-0.33	0.39	18	-0.5	0.45	24	9.3%	0.17 [-0.08, 0.42]	2018
**Total (95% CI)**			**85**			**90**	**100.0%**	**0.06 [-0.02, 0.14]**	

Heterogeneity: Chi² = 0.87, df = 2 (P = 0.65); I² = 0%
Test for overall effect: Z = 1.45 (P = 0.15)

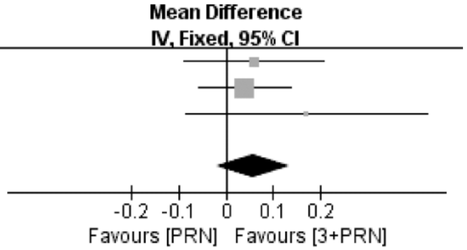

BCVA (logMAR)

b

Study or Subgroup	PRN Mean	PRN SD	PRN Total	3+PRN Mean	3+PRN SD	3+PRN Total	Weight	Mean Difference IV, Fixed, 95% CI	Year
Ito 2015	-250	252	25	-221	233	27	12.0%	-29.00 [-161.22, 103.22]	2015
Miwa 2017	-171	156	42	-207	155	39	45.5%	36.00 [-31.77, 103.77]	2017
Bayat 2018	-153	175	18	-243	160	24	19.7%	90.00 [-13.12, 193.12]	2018
Tang 2022	-300	217	34	-298	187	35	22.8%	-2.00 [-97.70, 93.70]	2022
**Total (95% CI)**			**119**			**125**	**100.0%**	**30.17 [-15.56, 75.90]**	

Heterogeneity: Chi² = 2.53, df = 3 (P = 0.47); I² = 0%
Test for overall effect: Z = 1.29 (P = 0.20)

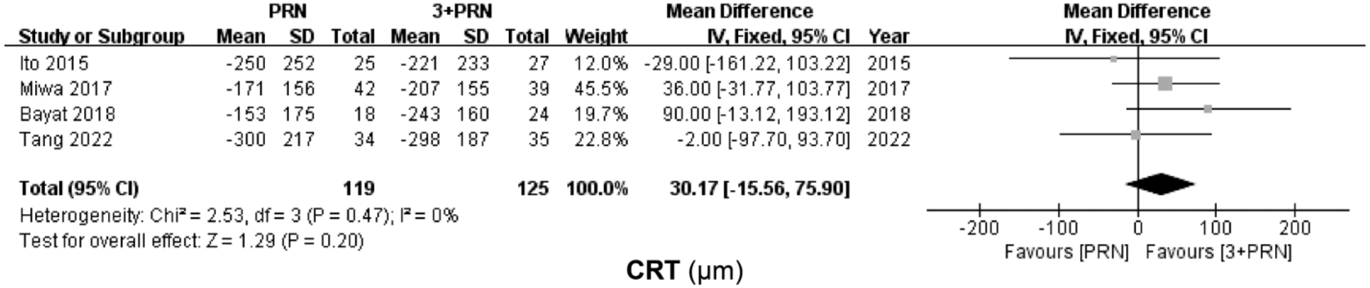

CRT (μm)

c

Study or Subgroup	PRN Mean	PRN SD	PRN Total	3+PRN Mean	3+PRN SD	3+PRN Total	Weight	Mean Difference IV, Random, 95% CI	Year
Ito 2015	2.1	0.8	25	4.3	1.4	27	28.9%	-2.20 [-2.81, -1.59]	2015
Miwa 2017	3.8	1.8	42	4.6	1.4	39	27.3%	-0.80 [-1.50, -0.10]	2017
Bayat 2018	2.8	1.6	18	4.2	1.3	24	23.5%	-1.40 [-2.30, -0.50]	2018
Tang 2022	4.2	2.4	34	5	2.2	35	20.4%	-0.80 [-1.89, 0.29]	2022
**Total (95% CI)**			**119**			**125**	**100.0%**	**-1.35 [-2.09, -0.60]**	

Heterogeneity: Tau² = 0.40; Chi² = 10.43, df = 3 (P = 0.02); I² = 71%
Test for overall effect: Z = 3.53 (P = 0.0004)

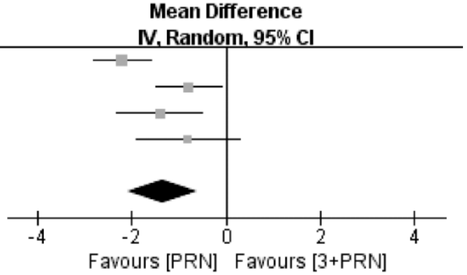

Total No. of IVT

Additional file 1. Sensitivity analysis presented as forest plots. The mean differences in (a) best-corrected visual acuity by logMAR scale, (b) central retinal thickness, and (c) total number of intravitreal injections with 95% confidence intervals comparing PRN group to 3+PRN group.
